# Chest Pain in Vain: Spontaneous Portal Vein Thrombosis

**DOI:** 10.7759/cureus.119284

**Published:** 2026-11-07

**Authors:** Simonas Makstutis, Suzy Collins

**Affiliations:** 1 Acute Internal Medicine, Stockport NHS Foundation Trust, Manchester, GBR

**Keywords:** chest pain, diagnostic imaging limitations, mesenteric vein thrombosis, portal vein thrombosis (pvt), splanchnic vein thrombosis

## Abstract

A 53-year-old man with a patient-reported history of ultrasound-diagnosed fatty liver disease presented with sudden-onset severe chest pain and a brief loss of consciousness, followed by progressive abdominal pain and a single episode of bloody diarrhoea. Admission investigations approximately 24 hours after the chest pain episode showed a D-dimer of 14,502 µg/L and high-sensitivity troponin I below 3 ng/L. An arterial-phase computed tomography (CT) aortogram showed no aortic dissection but did not establish portal venous patency. Following specialist thoracic radiology review, the aortogram was considered satisfactory to exclude pulmonary embolism down to the segmental level, and dedicated pulmonary angiography was not performed. Portal venous-phase CT performed 72 hours after the aortogram demonstrated extensive portal, superior mesenteric, and splenic vein thrombosis, with equivocal caecal and ascending colonic changes. Symptoms improved during anticoagulation. Repeat CT three months later showed resolution of thrombosis, with all three affected veins patent.

This case illustrates the importance of selecting an imaging protocol appropriate to the evolving clinical presentation. An unrevealing arterial-phase examination did not exclude portal venous thrombosis. The relationship between the thrombosis and the initial chest pain and collapse remains uncertain, as does the contribution of the patient's reported fatty liver disease.

## Introduction

Portal vein thrombosis (PVT), or more broadly splanchnic vein thrombosis, is a common complication of chronic liver disease or intra-abdominal inflammation, yet remains relatively rare in the general population [1]. Approximately four out of 100,000 men and two out of 100,000 women develop PVT every year [2]. An underlying predisposing condition is identified in approximately 70% of non-cirrhotic patients, although some cases remain unexplained after investigation [3]. In most cases of PVT, there is at least one element of Virchow's triad at play: endothelial injury, stasis, or a hypercoagulable state, often associated with chronic liver disease, intra-abdominal inflammation, or systemic thrombophilia [1,4]. Left untreated, PVT can lead to portal hypertension and associated complications such as splenomegaly and variceal bleeding [1,4]. This report describes PVT identified during the investigation of sudden-onset chest pain and collapse, followed by abdominal pain and bloody diarrhoea. The relationship between the thrombosis and the initial chest pain and collapse remains uncertain.

This case report was previously presented as a poster at the 2026 Society for Acute Medicine Glasgow conference on October 8, 2026.

## Case presentation

A 53-year-old man presented following an episode of sudden-onset severe stabbing and tearing chest pain almost 24 hours earlier. A few minutes after the onset of chest pain, the patient collapsed with a brief episode of total loss of consciousness and recovered spontaneously. The initial severe chest pain eased several minutes later, but the patient then started noticing abdominal pain, which gradually got worse. Over the next 12 hours, the abdominal pain became constant, generalised, and more severe. Overnight, he had several episodes of diarrhoea, including a single episode containing fresh blood. The patient attended our Emergency Department the following day, complaining of ongoing chest and abdominal pain. He was given 10 mg of intravenous morphine for symptom control before being referred to the Medical Same Day Emergency Care (SDEC) unit with chest pain as the primary complaint.

The patient reported a previous ultrasound diagnosis of fatty liver disease at another hospital, described on admission as metabolic dysfunction-associated steatotic liver disease (MASLD). No documentation of elastography or other formal fibrosis staging was available. He had no other known chronic diseases. He was not taking regular medication, had never smoked, did not drink alcohol, and was completely independent with activities of daily living.

The patient was reviewed by a senior doctor in Medical SDEC, who remained concerned about a serious underlying condition despite vital parameters being within the normal range. Abdominal examination demonstrated generalised tenderness with involuntary guarding but no rebound tenderness. Initial investigations were performed approximately 24 hours after the chest pain episode. The electrocardiogram showed sinus rhythm at 98 beats per minute, with no ischaemic changes or features suggestive of right heart strain (Figure 1). Blood results were largely unremarkable apart from a markedly raised D-dimer, mildly elevated C-reactive protein, and marginally deranged liver enzymes (Table 1). High-sensitivity troponin I was below 3 ng/L approximately 24 hours after the chest pain episode, and serial measurements were not performed. The patient remained on cardiac monitoring overnight, with no arrhythmias identified.

**Figure 1:**
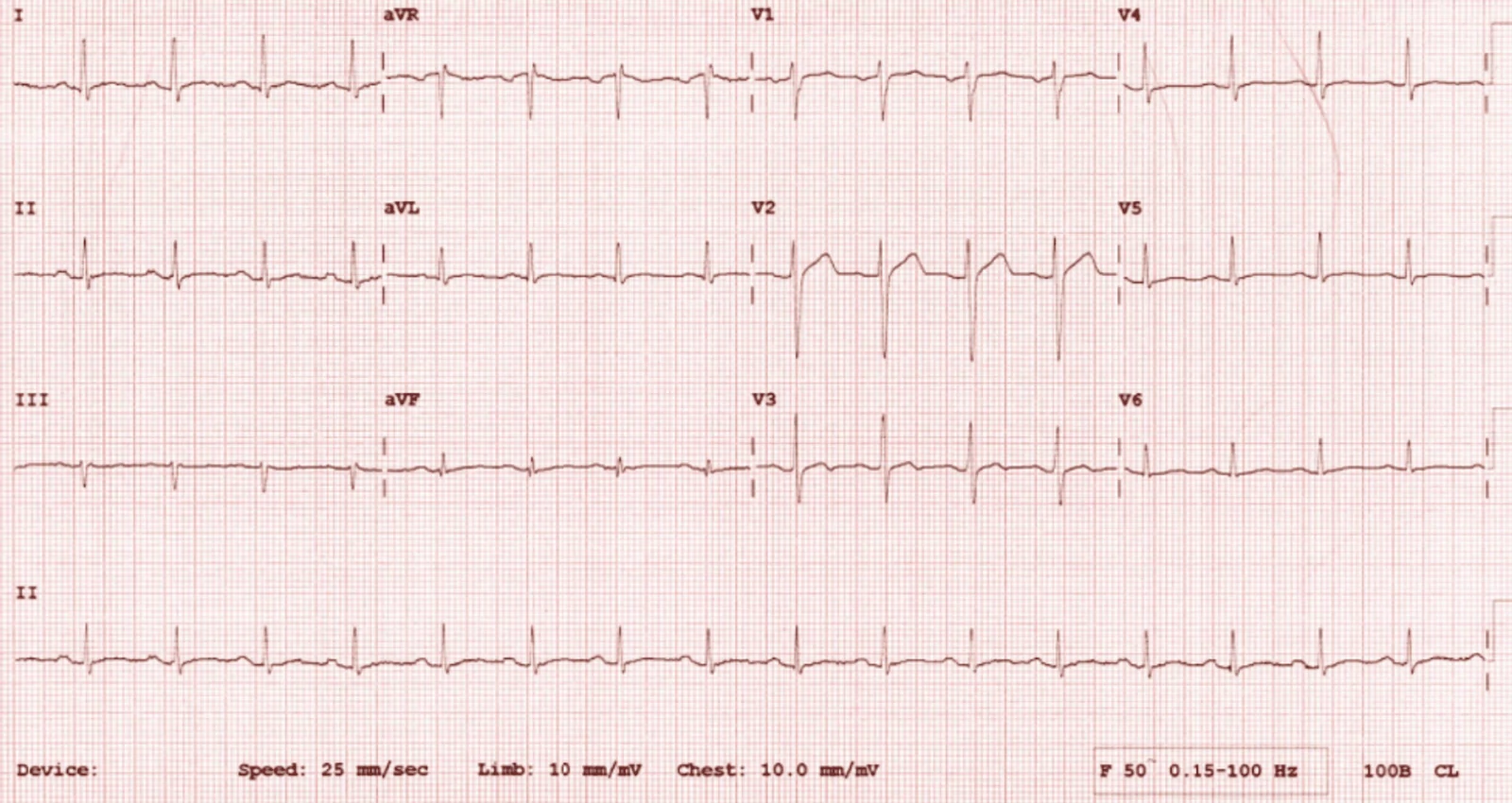
Admission electrocardiogram Scanned reproduction of the patient’s original printed 12-lead electrocardiogram demonstrating normal sinus rhythm at 98 beats per minute, with no ischaemic changes or features suggestive of right heart strain.

**Table 1:** Blood test results on presentation ↑ indicates values above the laboratory reference interval; — indicates that no reference interval was provided. Reference intervals are those reported by the laboratory. Blood gas measurements were obtained from a venous sample.

Test (unit)	Result	Reference interval
White cell count (×10⁹/L)	8.4	3.7–11.0
Haemoglobin (g/L)	144	130–180
Platelets (×10⁹/L)	195	150–450
Total bilirubin (µmol/L)	40 ↑	1–21
Alanine aminotransferase (U/L)	59 ↑	0–45
Alkaline phosphatase (U/L)	64	20–130
Gamma-glutamyl transferase (U/L)	37	12–64
Prothrombin time (s)	11.7	9.4–12.5
International normalised ratio	1.1	—
D-dimer (µg/L)	14,502 ↑	42–530
Sodium (mmol/L)	139	133–146
Potassium (mmol/L)	4.3	3.5–5.3
Urea (mmol/L)	4.4	2.5–7.8
Creatinine (µmol/L)	97	62–115
Estimated glomerular filtration rate (mL/min/1.73 m²)	73	—
C-reactive protein (mg/L)	44.8 ↑	0.0–10.0
High-sensitivity troponin I (ng/L)	<3	0–58
pH	7.36	7.35–7.45
Bicarbonate (mmol/L)	28.2	22.0–29.0
Lactate (mmol/L)	1.0	0.6–2.0

Given the sudden-onset severe chest pain and collapse, the leading initial differential diagnoses included aortic dissection and pulmonary embolism. There was some concern regarding the abdominal pain, and abdominal imaging was considered, but it was felt that the origin of the problem was in the chest area. This presented a significant diagnostic challenge: arterial-phase computed tomography (CT) angiography is used to assess suspected aortic dissection [5], whereas a dedicated CT scan of the abdomen with a portal venous phase is better suited to assessing abdominal pathology such as PVT and its abdominal complications [6]. After discussion with a consultant radiologist, it was agreed that a CT aortogram would reveal the most information in this presentation. It would not include venous-phase imaging, which could be performed separately at a different time if still indicated.

The CT aortogram was reported as showing no aortic dissection or other acute abnormality. The thoracic section demonstrated a normal ascending and descending aorta (Figure 2). Portal and splenic venous patency could not be reliably assessed on this arterial-phase acquisition (Figure 3). Retrospective review could not establish whether thrombosis was already present.

**Figure 2:**
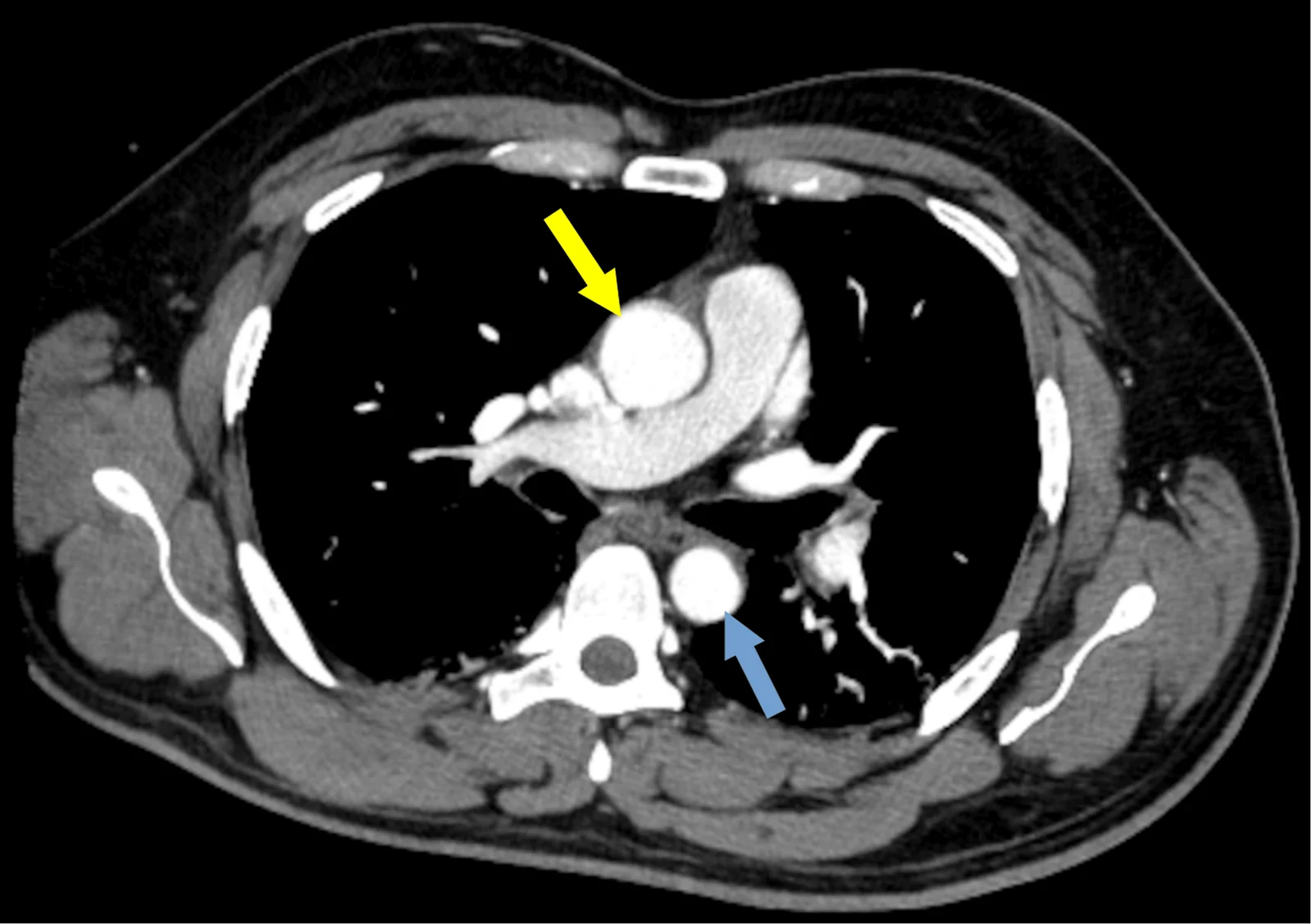
Thoracic section of the admission aortogram Axial section from the arterial-phase computed tomography aortogram showing the ascending thoracic aorta (yellow arrow) and descending thoracic aorta (blue arrow), neither of which shows evidence of aortic dissection.

**Figure 3:**
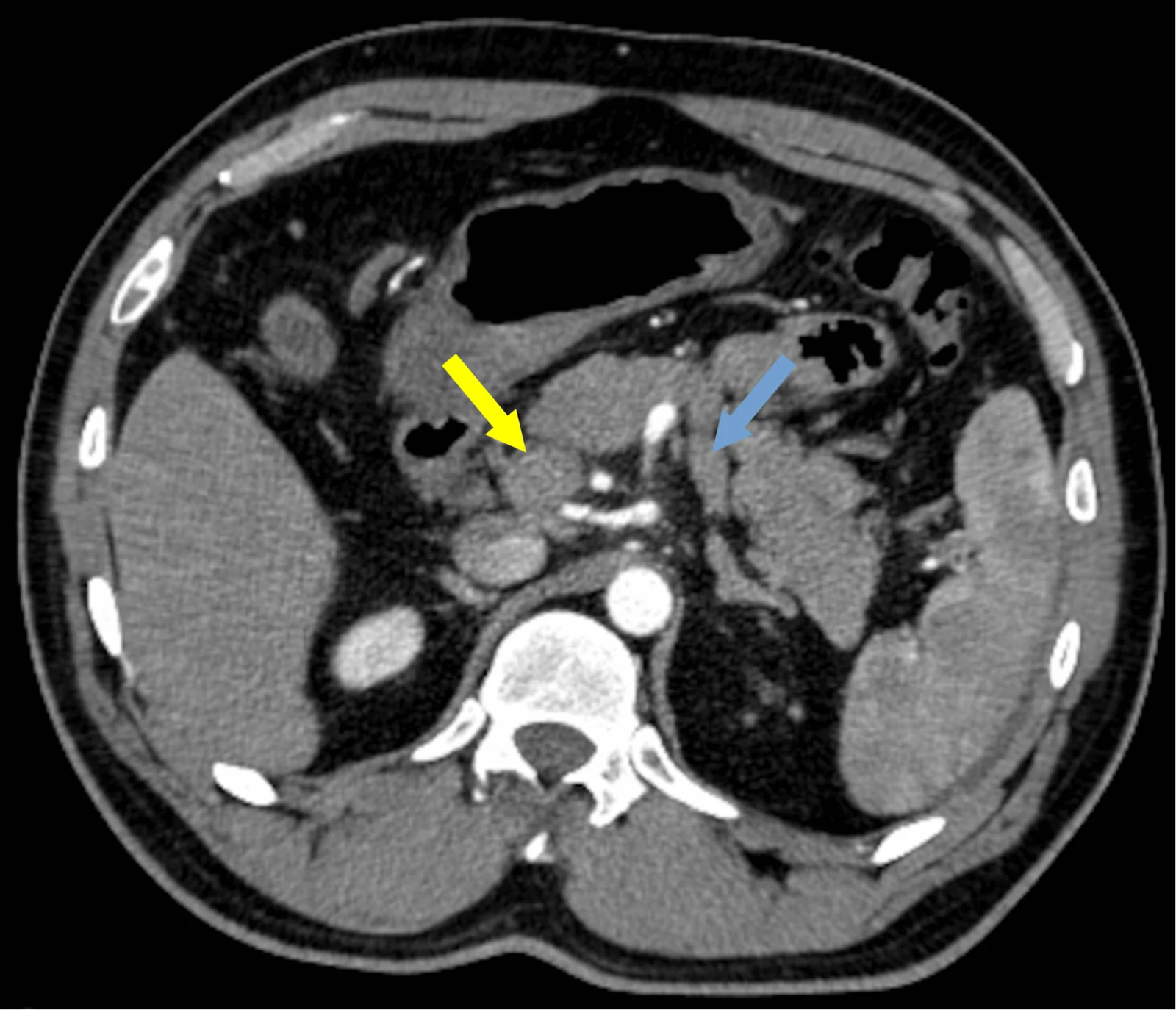
Abdominal section of the admission aortogram Axial section from the arterial-phase computed tomography aortogram. The yellow arrow indicates the portal vein; the blue arrow indicates the splenic vein. Thrombosis is not apparent on this arterial-phase image, but due to the arterial phase of the scan and insufficient contrast enhancement of the veins, it does not establish venous patency either. Subsequent portal venous-phase CT demonstrated venous filling defects.

The patient was admitted without a clear diagnosis. Despite the unrevealing aortogram, clinical suspicion of pulmonary embolism remained high. Therapeutic low-molecular-weight heparin (LMWH), as enoxaparin 120 mg (1.5 mg/kg) subcutaneously once daily, was started pending a dedicated CT pulmonary angiogram (CTPA). Broad-spectrum antibiotics were started empirically to cover possible intra-abdominal infections, such as cholangitis or colitis.

On abdominal reassessment the following day, the abdomen was soft, guarding had resolved, and pain was improving. The bloody diarrhoea did not recur during therapeutic LMWH, and symptoms continued to improve while investigations proceeded.

Following verbal discussion with a specialist thoracic radiologist, it was documented that the aortogram was satisfactory to exclude pulmonary embolism down to the segmental level. A dedicated CTPA was therefore not performed. Further technical details of pulmonary arterial enhancement and image quality were not recorded.

Abdominal ultrasound performed 48 hours after the initial aortogram showed an echogenic liver consistent with steatosis, without another acute abnormality. Colour Doppler assessment of the portal and mesenteric venous circulation was not performed.

A dedicated portal venous-phase CT of the abdomen performed 72 hours after the initial aortogram demonstrated extensive thrombosis of the main portal vein, its right and left intrahepatic branches, the proximal splenic vein, and the superior mesenteric vein (SMV), as shown in Figures 4-6. The report stated that a thrombus occluded most of the portal vein at the confluence, at the level of the pancreatic head. No portal venous or portosystemic collaterals or splenomegaly were identified. The recent symptom onset and markedly raised D-dimer, together with the absence of identified collateral circulation, supported a clinical interpretation of recent PVT, although the precise thrombus age remained uncertain. The report also described equivocal thickening/oedema of the caecum and ascending colon, minimal pericolonic fat stranding, and no pericolonic collection. Bowel wall enhancement was not further characterised in the available report. In the setting of SMV thrombosis, these findings raised concern for possible venous congestion or early ischaemic change; bowel infarction was not established.

**Figure 4:**
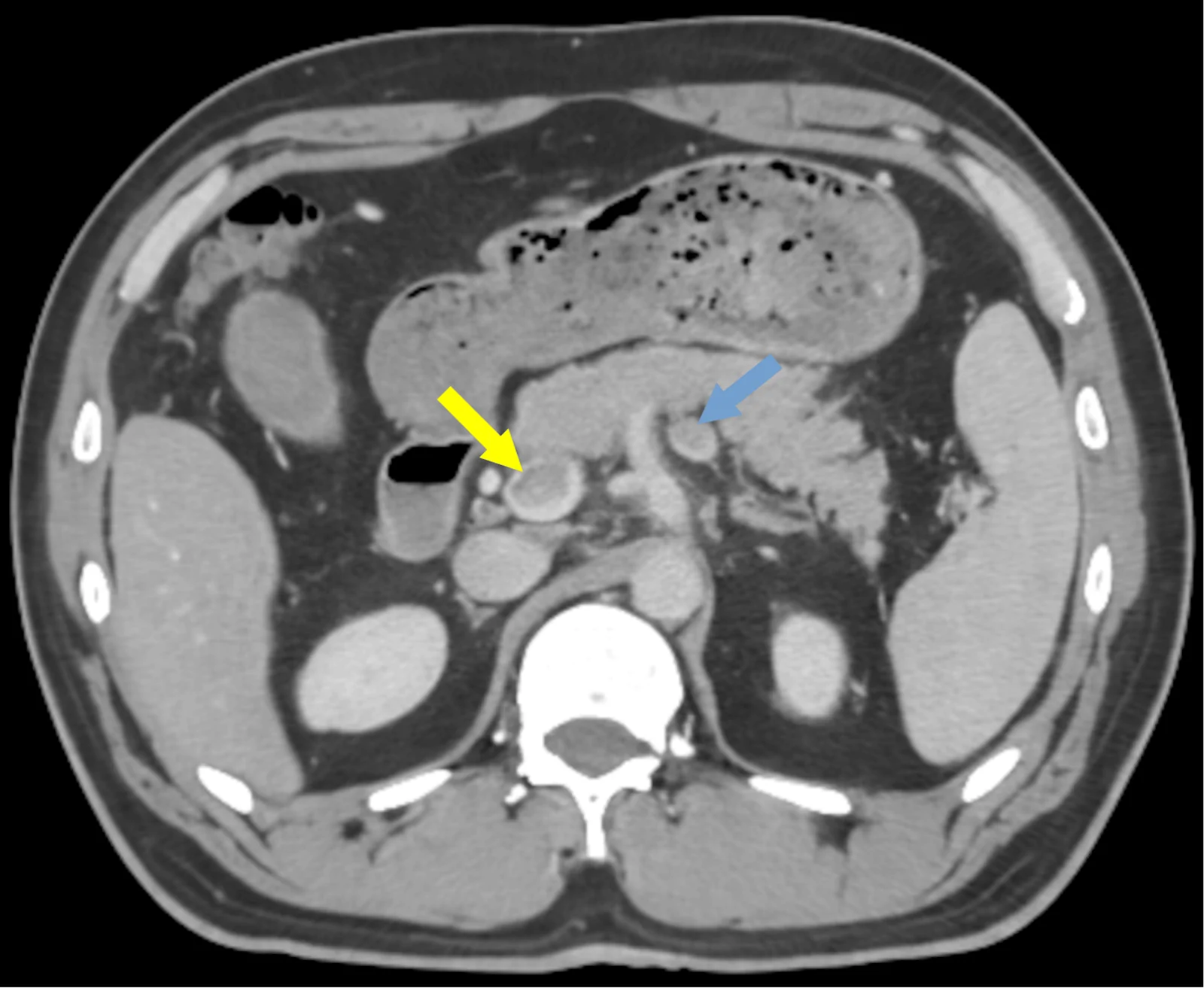
Portal vein and splenic vein thrombosis Axial section of contrast-enhanced computed tomography of the abdomen in the portal venous phase demonstrates filling defects in the portal vein (yellow arrow) and splenic vein (blue arrow) in keeping with thrombosis.

**Figure 5:**
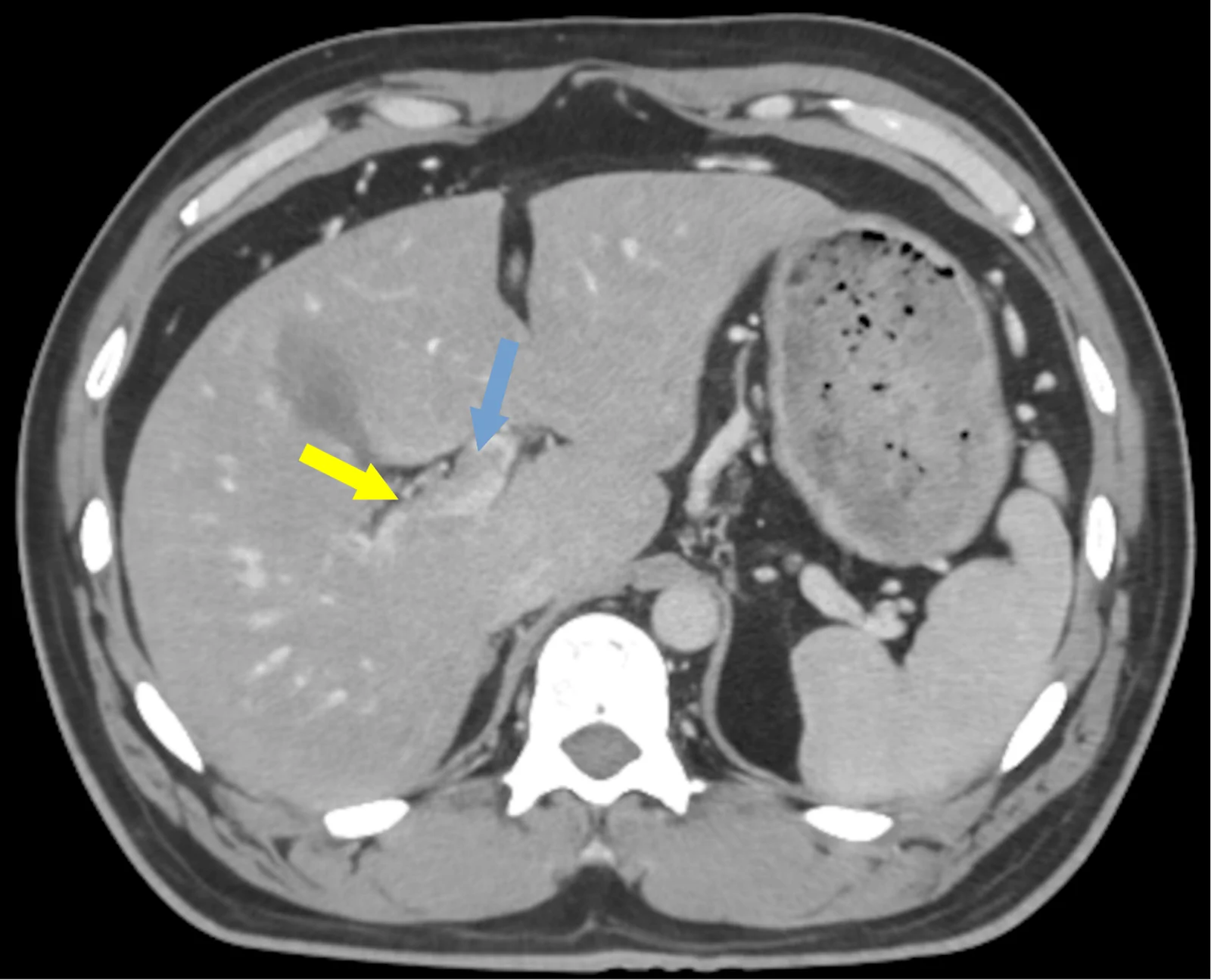
Intrahepatic portal vein thrombosis Axial section of contrast-enhanced computed tomography of the abdomen in the portal venous phase demonstrates thrombosis of the intrahepatic branches of the portal vein. The yellow arrow indicates thrombus in the right intrahepatic branch of the portal vein; the blue arrow indicates thrombus in the left intrahepatic branch.

**Figure 6:**
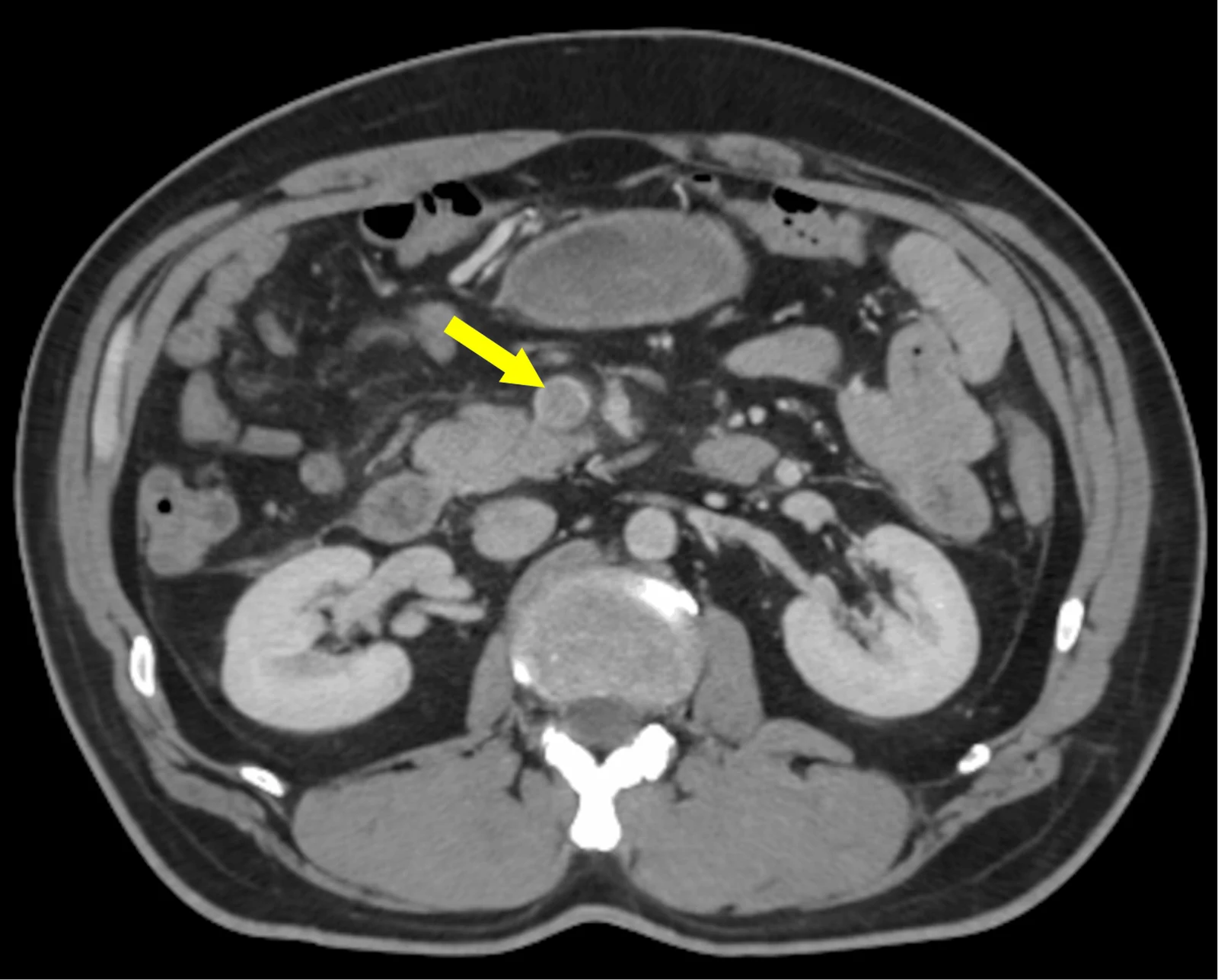
Superior mesenteric vein thrombosis Axial section of contrast-enhanced computed tomography of the abdomen in the portal venous phase demonstrates a filling defect in the superior mesenteric vein (yellow arrow), in keeping with thrombosis.

The initially reassuring aortogram, improved abdominal pain, resolution of guarding, and absence of further bloody diarrhoea influenced conservative management. The normal admission lactate provided additional reassurance, although it did not exclude bowel ischaemia. Care was transferred to gastroenterology; surgical review was not sought. No further cardiac investigations were performed after the abdominal pathology had been identified.

Gastroscopy was performed to assess for complications of portal hypertension. This was the patient's first endoscopy and showed severe portal hypertensive gastropathy (Figure 7), without varices. The duration and cause of the gastropathy could not be established, and pre-existing portal hypertension could not be excluded. Sigmoidoscopy, performed in view of the bleeding episode, was largely normal apart from small haemorrhoids. Sigmoidoscopy images were not available for inclusion.

**Figure 7:**
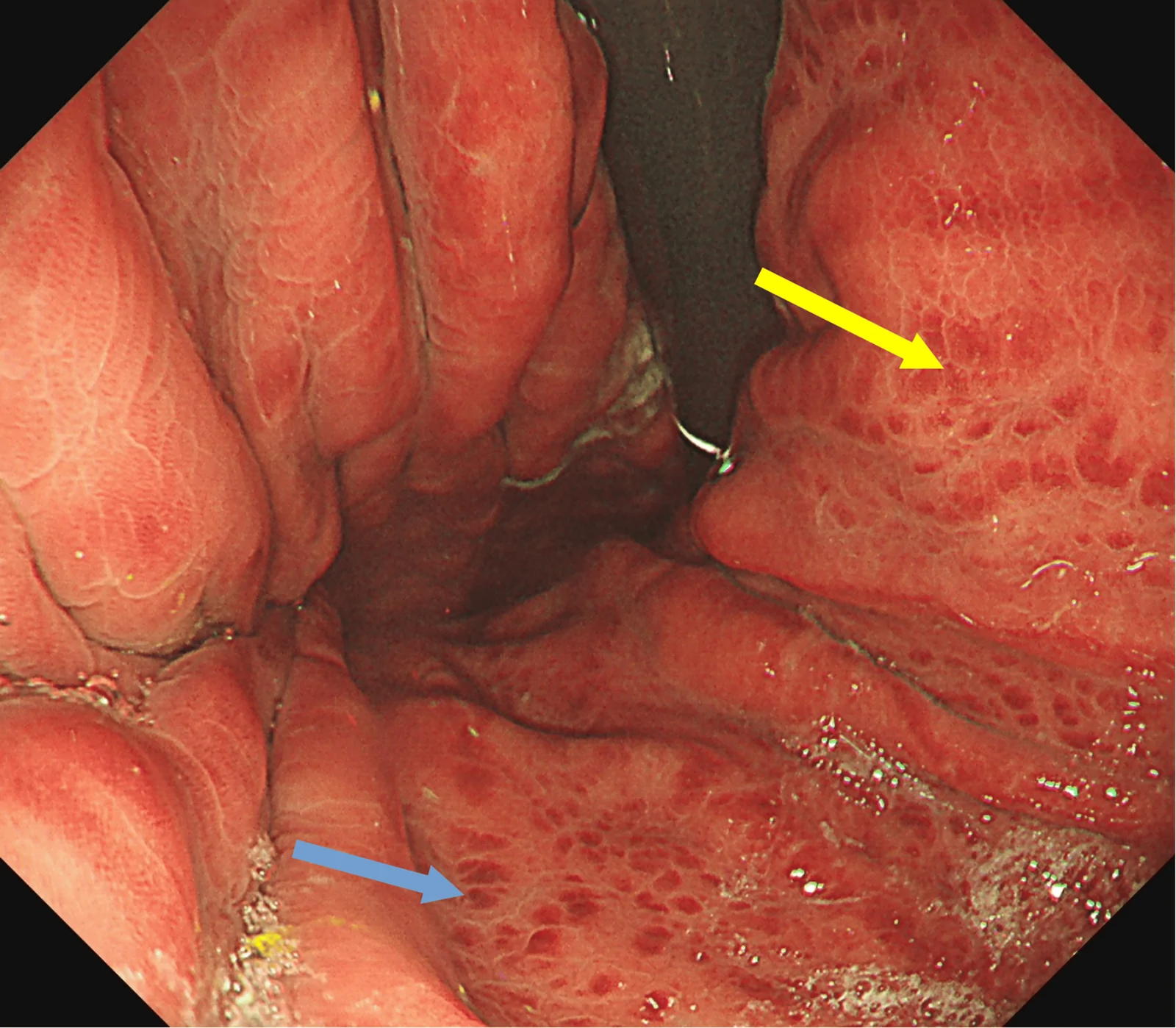
Portal hypertensive gastropathy Gastroscopic image of the stomach showing a reticulated, mosaic-like mucosal pattern (yellow arrow) and ectatic blood vessels (blue arrow), both features of severe portal hypertensive gastropathy.

Following discussion with haematology, the episode was considered apparently unprovoked on the initial assessment. The initial evaluation did not identify malignancy or a local inflammatory precipitant such as pancreatitis. The contribution of the reported fatty liver disease remained uncertain, and the aetiological evaluation was incomplete.

Initial investigations were negative for antinuclear antibodies, anticardiolipin antibodies, anti-β2-glycoprotein antibodies, factor V Leiden, and prothrombin G20210A mutations. Janus kinase 2 (JAK2) V617F, CALR, and MPL mutation testing was also negative. Lupus anticoagulant testing was reported as negative while the patient was receiving LMWH. The assay method, heparin-neutralisation details, and sampling time relative to the last LMWH dose were unavailable, so possible assay-dependent heparin interference could not be assessed.

The patient was switched to rivaroxaban 15 mg twice daily for three weeks, followed by 20 mg once daily, with a planned minimum treatment duration of six months. Protein C and protein S testing remained outstanding. Rivaroxaban can interfere with clot-based assays [7]. An outpatient haematology review was arranged to assess treatment duration and complete the evaluation. If interruption were considered safe, testing would be performed after an appropriate rivaroxaban-free interval agreed with the laboratory.

The patient's chest pain, abdominal pain, and diarrhoea resolved within several days of starting anticoagulation, and he was discharged with advice to continue oral treatment. A repeat CT three months later showed complete resolution of thrombosis, with the portal vein, splenic vein, and SMV all patent. The report did not comment on the previous caecal or ascending colonic changes.

At the latest documented follow-up, the patient remained on anticoagulation for a planned minimum of six months, with outpatient haematology review pending. No subsequent abdominal pain or lower gastrointestinal bleeding was documented. Portal hypertension had not yet been reassessed.

## Discussion

PVT, or more broadly splanchnic vein thrombosis, is uncommon in the general population and may not initially be considered in a patient presenting with sudden-onset chest pain. An underlying predisposing condition is identified in approximately 70% of non-cirrhotic cases [3]. Typical risk factors include liver cirrhosis, primary or secondary hepatobiliary malignancies, significant systemic infections, intra-abdominal inflammation, haematological malignancies, and thrombophilias [1].

Clinical presentations are varied: splanchnic vein thrombosis may cause abdominal pain, nausea, diarrhoea, gastrointestinal bleeding, or ascites but can also be asymptomatic or detected incidentally [8]. A similar case report described an idiopathic PVT in a 27-year-old woman who presented with two weeks of epigastric pain [9]. In contrast, our patient’s first symptoms were severe chest pain and collapse, followed by abdominal pain and bloody diarrhoea. This case illustrates that PVT can be encountered during investigation of an atypical chest-pain presentation, although the mechanism linking the thrombosis to the initial chest pain and collapse remains uncertain.

PVT can be recent or chronic. Patients with recent PVT may be asymptomatic or present with generalised abdominal pain, normal or mildly elevated transaminases, and features suggesting mesenteric ischaemia, such as diarrhoea or a raised lactate when mesenteric veins are involved. Chronic PVT may present with manifestations of underlying disease or complications of portal hypertension [1,8]. In this patient, SMV involvement and the equivocal bowel changes on CT provided a possible explanation for the abdominal pain and bloody diarrhoea.

A recent meta-analysis estimated a PVT prevalence of 8.5% in the included fatty liver disease cohorts, although these predominantly involved more advanced liver disease [10]. MASLD is common, with an estimated prevalence of approximately 25% in Western Europe [11]. Our patient reported a previous ultrasound diagnosis of fatty liver disease, supported by the echogenic appearance of the liver during this admission, but formal fibrosis staging was not available. The pooled prevalence cannot be interpreted as this patient's individual risk, and the contribution of hepatic steatosis to the thrombosis remains uncertain. Severe portal hypertensive gastropathy at his first endoscopy did not establish the duration of portal hypertension or prove cirrhosis or chronic PVT. The absence of identified collaterals or splenomegaly did not exclude pre-existing portal hypertension.

This patient underwent several imaging modalities before a diagnosis was established. The initial arterial-phase CT aortogram did not establish portal venous patency, and the routine abdominal ultrasound was performed without colour Doppler assessment. Ultrasound with Doppler assessment of portal venous flow is an important diagnostic modality, with a reported sensitivity of 89-93%, although performance depends on factors including operator experience, obesity, and ascites [4,6]. A routine abdominal ultrasound without assessment of portal venous flow should not be taken as excluding PVT.

Portal venous-phase CT can offer similar sensitivity while assessing extension into the mesenteric or splenic veins and associated bowel complications [6]. It carries risks from iodinated contrast and radiation exposure [6]. Adequate portal venous opacification is important for identifying intraluminal filling defects, and multiphase imaging may provide additional information [6]. In this case, the portal venous-phase examination established the diagnosis despite an improving clinical course. The interval of 72 hours between scans does not establish when the thrombus developed, since retrospective review of the initial arterial-phase study remained indeterminate.

Evaluation for underlying prothrombotic conditions remains important even when initial investigations are negative. Assessment should consider local inflammatory or malignant causes, inherited and acquired thrombophilia, and myeloproliferative neoplasms; a normal blood count does not exclude the latter, and JAK2 V617F mutation testing may be informative [3,4]. The timing and interpretation of thrombophilia testing must account for the acute thrombotic event and anticoagulant therapy [3,7]. In this patient, negative initial investigations, including mutation testing, did not amount to exclusion of every prothrombotic cause. Outstanding protein C and protein S testing supports continued haematology follow-up and a provisional classification as apparently unprovoked.

Complications of PVT include portal hypertension, which can lead to the formation of varices in the gastrointestinal tract and subsequent variceal bleeding. In longstanding chronic PVT, collateral veins can compress the bile ducts, causing portal cholangiopathy that may present with obstructive jaundice, pruritus, cholecystitis, or cholangitis [1].

Treatment of acute non-cirrhotic PVT is mainly anticoagulation for a minimum of six months, with long-term anticoagulation considered in unprovoked cases. In chronic PVT, decisions about anticoagulation should be individualised according to cirrhosis status and the risks of recurrent thrombosis and bleeding. Management also includes treatment of portal hypertension-related complications and any underlying disease [3,4]. Some retrospective studies have shown that partial recanalisation of the portal vein can be achieved in 63-93% of patients when they are treated with anticoagulants. Moreover, complete recanalisation is possible in 34-45% of non-cirrhotic patients [1].

A prospective study evaluated rivaroxaban in symptomatic non-cirrhotic splanchnic vein thrombosis using 15 mg twice daily for three weeks followed by 20 mg once daily. Complete recanalisation was documented in 47.3% of patients at three months, with major bleeding reported in 2.1%; the single-arm design limits comparison with other anticoagulants [12]. These findings provide context for the complete resolution observed in our patient after three months.

In patients with persistent symptoms, intestinal ischaemia, or refractory portal hypertensive complications despite anticoagulation, specialist options include portal vein recanalisation, transjugular intrahepatic portosystemic shunt, and selected thrombectomy or thrombolysis. These interventions require individualised assessment in experienced centres because of their procedural and bleeding risks [4,6].

The improving pain, resolution of guarding, and absence of further bloody diarrhoea supported conservative management; the normal admission lactate provided additional reassurance, although it did not exclude early bowel ischaemia. As a result, no surgical review was obtained. Without further characterisation of bowel wall enhancement, the CT findings and clinical course could not definitively distinguish venous congestion from early ischaemic change.

## Conclusions

This case highlights the importance of reconsidering the imaging protocol when abdominal symptoms remain unexplained after an arterial-phase CT examination. Portal venous-phase CT identified extensive splanchnic vein thrombosis and equivocal bowel changes that had not been demonstrated on the initial aortogram. The arterial-phase examination did not establish venous patency. The relationship between the thrombosis and the initial chest pain and collapse remains uncertain, as does the contribution of hepatic steatosis. The principal diagnostic lesson is to select imaging appropriate to the evolving presentation while continuing evaluation for underlying prothrombotic conditions.
